# Evaluation of Wearable Cameras for Monitoring and Analyzing Calf Behavior: A Preliminary Study

**DOI:** 10.3390/ani11092622

**Published:** 2021-09-07

**Authors:** Tomoko Saitoh, Yuko Kato

**Affiliations:** Field Center of Animal Science and Agriculture, Obihiro University of Agriculture and Veterinary Medicine, Obihiro 080-8555, Japan; yuko.kato.1234@gmail.com

**Keywords:** ethology, calf, behavioral observations, wearable camera, standing, lying, rumination, feeding

## Abstract

**Simple Summary:**

Owing to the small size and lightweight of wearable cameras, they do not affect cattle behavior when attached to their bodies. Thus, this study aimed to evaluate the suitability of wearable cameras for monitoring and analyzing calf behavior. We conclude that wearable cameras are suitable for observing calf behavior, particularly their posture (standing or lying), as well as their ruminating and feeding behaviors.

**Abstract:**

Understanding cattle behavior is important for discerning their health and management status. However, manual observations of cattle are time-consuming and labor-intensive. Moreover, during manual observations, the presence or position of a human observer may alter the normal behavior of the cattle. Wearable cameras are small and lightweight; therefore, they do not disturb cattle behavior when attached to their bodies. Thus, this study aimed to evaluate the suitability of wearable cameras for monitoring and analyzing cattle behavior. From December 18 to 27, 2017, this study used four 2-month-old, group-housed Holstein calves at the Field Science Center of the Obihiro University of Agriculture and Veterinary Medicine, Japan. Calf behavior was recorded every 30 s using a wearable camera (HX-A1H, Panasonic, Japan) from 10:00 to 15:30 and observed directly from 11:00 to 12:00 and 14:00 to 15:00. In addition, the same observer viewed the camera recordings corresponding to the direct observation periods, and the results were compared. The correlation coefficients of all behavioral data from direct and wearable camera video observations were significant (*p* < 0.01). We conclude that wearable cameras are suitable for observing calf behavior, particularly their posture (standing or lying), as well as their ruminating and feeding behaviors.

## 1. Introduction

Understanding cattle behavior is important in discerning their health and management status [1]. Behavioral observations are an effective means of understanding the health condition and feeding management status of dairy cows. However, manual observation of cattle is time-consuming and labor-intensive [2]. The limitations of manual observation include personnel training, subjectivity, and brevity [3]. In addition, inter-observer reliability is affected by observer experience when observing multiple animals using many observers [4], and observer expectations may invalidate the subjective recording of behavior [5]. It is common to observe behavior using recordings from a fixed-point video camera [6]; however, it is difficult to observe free-ranging animals because the observational accuracy can be reduced by the camera’s field of view and blind spots.

Automatic behavioral measurement devices using various sensors are being researched and developed. The first commercially used sensors were developed for the detection of reproductive behavior (mounting and standing); managing reproduction is directly related to farm profit. These have been studied since the 1990s [7] and include applications for electronic estrus detection [8], field evaluation of activity, meters for detecting cows in estrus in a large pasture-grazed dairy farm [9], assessment of an accelerometer system for the detection of estrus [10], and comparisons of automated and visual measurements of estrous behavior [11].

There are also sensors for detecting eating and rumination behaviors because the amount of feed a cow consumes and ruminates is directly connected to milk and meat production. These include pressure sensors mounted on the noseband of the halter to monitor eating and ruminating [12]; a pressure sensor and accelerometer attached to the noseband of a halter are commercially available for automatically measuring the position of the head, thus detecting behaviors such as rubbing, feeding, drinking water, and moving [13,14], Additionally, a bolus sensor with support vector machine has also been developed [15].

Furthermore, ear tag sensors can predict the start of calving in dairy cows [16], and body temperature [17] and lameness detection sensors [18] have been developed. Some sensors have adequate accuracy, for example, the heat stress sensors have a high variability [19], and the accelerometer is a promising monitoring system for feeding behavior [20]. The ear-tag sensor accurately monitors the rumination and eating behavior of grazing dairy cattle; however, active behaviors may be more difficult for the sensor to record than other behaviors [21].

Data from various sensors that automatically monitor cow behavior are collected as numerical values, with no information on the behavior itself. Various sensors are used for cattle, but only a few examples of their use in calves exist [22,23,24,25,26,27]. As calves present low productivity, few studies have assessed calf behavior or utilized behavior as an indicator for health management in dairy farming. However, the feeding and raising periods of dairy heifers can last for 2 years or more. Raising calves is an investment in future milk production, and production costs are significant [28].

Therefore, in the present preliminary study, we utilized a behavioral observation method by attaching a wearable camera to calves. Several studies have reported their application in goats [29] and sea turtles [30], but there is no study on the daily behavioral time budgets. The camera was chosen based on its small size and its ability to be worn on the animal’s body. As the wearable camera is small and lightweight, it does not burden the cow or restrict its behavior when attached. In addition, various camera models are commercially available and can be readily obtained at low costs. Recently, changes in the size and weight of the battery and recording media have enabled the size of the body camera to be reduced. Therefore, they can be used to observe the behavior of cows because it is possible to obtain high-resolution images. Although behavioral data are limited by the nature of the sensor, various types of behavioral data can be gathered by observing the behavior on images obtained using a wearable camera. Recording videos of cattle for behavioral observation using fixed-point cameras is associated with many problems. For example, identification is difficult without markings and there is a limitation of the recording field owing to the field of view of the cameras and blind spots. Videos recorded using a wearable camera are valuable because the camera is attached to the animals and moves with them, circumventing identification problems and allowing a closer look for targeted behavior. Additionally, because the videos can be viewed offline by the observer, it is possible to avoid the time constraints of real-time observations. Moreover, videos can be observed indoors in the comforting surroundings of a laboratory.

Video summarization using the machine-learning technology (AI) is an important technique of video analysis that has been steadily developing. The main approaches related to video summarization are generally divided into two parts [31]. One part is a static video summary, extracting a series of frames representing the video’s subjective contents. The other part is dynamic video skimming, composed by concatenating short video segments.

Automatic identification of a specific behavior of cattle from a video with machine-learning technology will greatly contribute to the health management of cattle and to the improvement of the barn environment. However, it first needs to be ascertained (by humans) if the recordings from wearable cameras can be used for automatic analysis. If it is confirmed that the images obtained from wearable cameras can accurately record the behavior of calves, then the videos can be used for automatic analyses using AI. In this study, we aimed to verify the possibility of recording the targeted behavior by a wearable camera to the exact degree of accuracy obtained by direct human visual observation.

## 2. Materials and Methods

### 2.1. Experimental Period and Location

**Figure 1:**
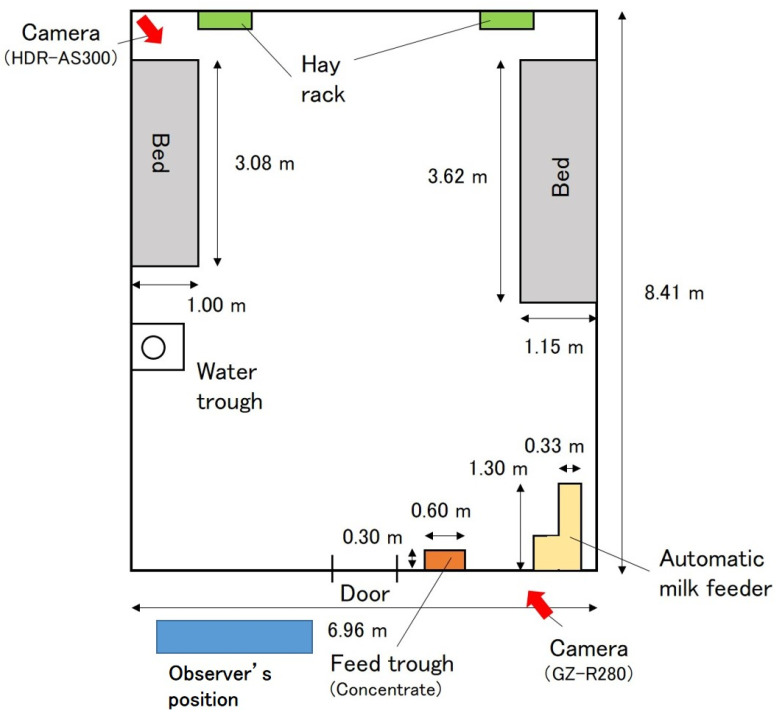
Barn layout where calves, fitted with wearable cameras for observing juvenile bovine behavior, were kept during the study. The position and direction of the fixed cameras are indicated by the red arrows.

The experimental procedures complied with the Guide for the Care and Use of Agricultural Animals of the Obihiro University of Agriculture and Veterinary Medicine. All methods were carried out in accordance with the university regulations on the Management and Operation of Animal Experiments (accepted No. 18–80). The study was conducted from 18–27 December 2017, in a calf barn with an automatic feeding system at the Field Science Center of the Obihiro University of Agriculture and Veterinary Medicine, Japan. Figure 1 shows the layout of the barn and the position and direction of the fixed cameras. The bed was covered with straw and sawdust. In the barn, cleaning work, such as changing the bedding, was carried out daily for approximately 30 min from approximately 10:00.

### 2.2. Test Calves

Four female Holstein calves, approximately 2-months-old, were observed, and their behavior was recorded (Table 1). The calves were well-adapted to the barn environment. As a result of management practices, up to three other calves (that were not part of the observation) were periodically kept in the barn. Consequently, the calves were kept in a group of four to seven during the experiment. On the day of behavioral observation, hay and concentrate were provided ad libitum to three of the four calves because they were already weaned. The fourth (unweaned) calf was fed using an automatic feeder. In addition, the calves were habituated to wear a wearable camera using a weighted halter once from 9:00 to 18:00 before observation.

### 2.3. Wearable Camera and Attachment

An HX-A1H camera (Panasonic, Osaka, Japan) was used in this experiment. The recording pixels were set to 280 × 720, and the frame rate was 30 fps. In addition, a wide mode with a view angle of approximately 150° was set to obtain a wide picture of the calf’s mouth. The use of a rechargeable mobile battery (3000 mAh) in addition to the main battery enabled longer recordings to be obtained. The mobile battery was replaced at approximately 13:00 to ensure the recording was completed.

**Figure 2:**
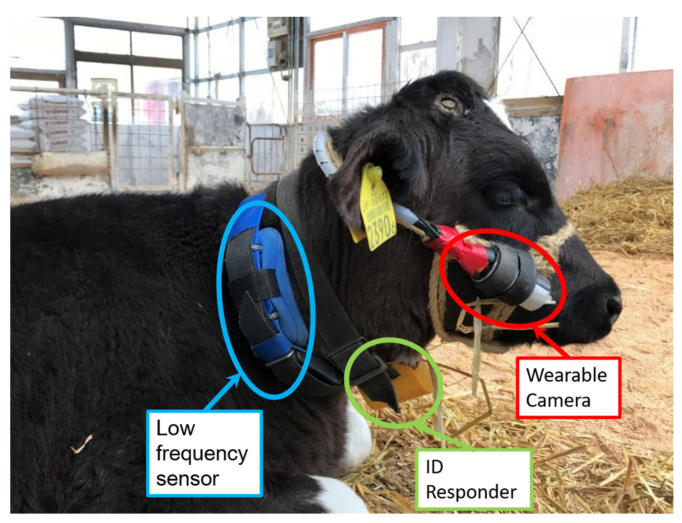
The halter and camera attached to a calf during the study investigating the suitability of wearable cameras for observing juvenile bovine behavior. The wearable camera is shown in the red circle. ID responder shown in the green circle is for automatic milk feeder. The low-frequency sensor shown in blue is for obtaining rumination data for another experiment, the results of which are not presented in this paper.

The wearable camera was placed in a protective case and fixed to the calf’s right cheek with a commercially available calf halter (Figure 2). The mobile battery was attached to the left cheek. The halter set with the fixed wearable camera weighed approximately 344 g (43 g for the wearable camera, 75 g for the mobile battery, and 226 g for the halter and wiring cables).

### 2.4. Behavioral Observations

Behavioral recordings by wearable cameras were made from 10:00 to 15:30, when it was possible to observe behavior without artificial lighting. In addition, to aid observations of visual behavior, two fixed-point cameras (HDR-AS300, SONY, Tokyo, Japan and GZ-R280, JVC, Kanagawa, Japan) were each installed in a position from which the entire barn could be recorded (Figure 1).

Direct observations were performed for a total of 2 h, from 11:00 to 12:00 and 14:00 to 15:00, for one animal per day. Animals were observed by an individual trained in behavioral observations at a position that was unlikely to affect the behavior of the calf. The behavior was recorded by instantaneous sampling every 30 s; posture and behavior definitions are shown in Table 2 and Table 3.

If any behavior was difficult to distinguish visually, the observer reviewed the recording from the fixed-point camera after the observation.

For behavioral observations using fixed camera recordings, the same observer played the recordings for the same period as the direct observations on a personal computer and registered the behavior in the same manner as reported for the direct observations. Camera recording observations were performed in January 2018. The observer was allowed to pause and replay the recordings during observation.

### 2.5. Statistical Analysis

The interrater reliability between the recorded behavioral data (behavior of every 30 s) from the direct and wearable camera video observations was calculated for each calf using Cohen’s kappa by BellCurve for Excel in Microsoft Excel, 2016. In addition, Cohen’s kappa was used to compare categorical data collected from two different methods [32]. This enabled us to determine the level of agreement between the two observation methods.

## 3. Results

### 3.1. Characteristics of Confirmed Behaviors

**Figure 3:**
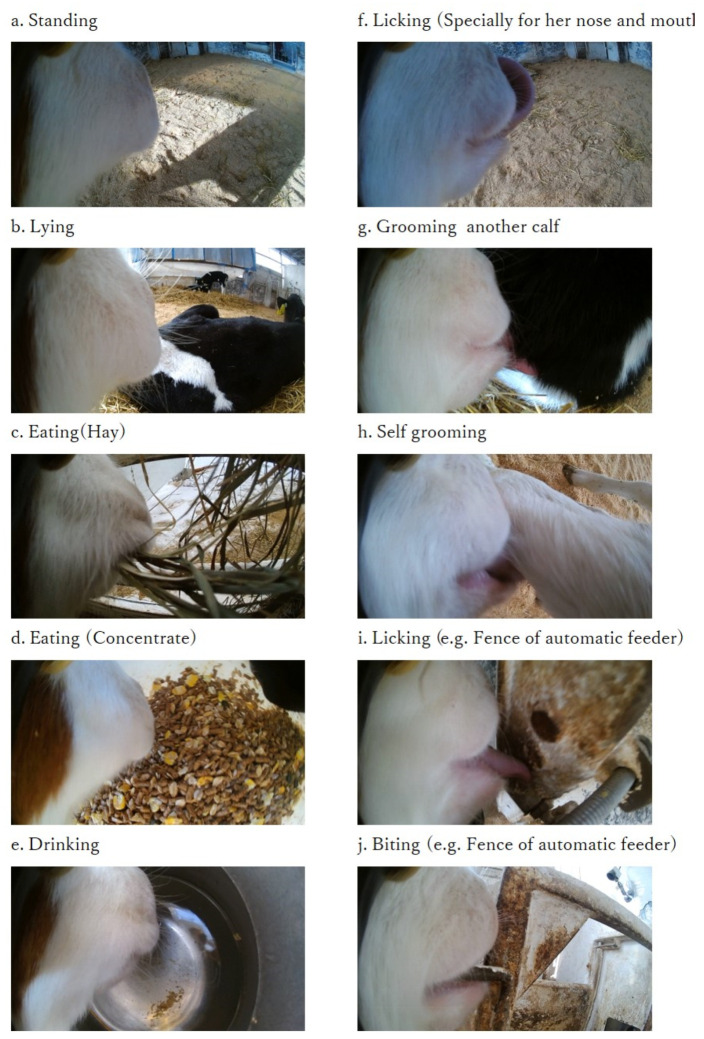
Representative images (calf No. 876) captured using a wearable camera for monitoring juvenile bovine behavior. The camera was fixed to the halter on the right cheek of the calf. Titled behaviors were recorded.

Behaviors could be confirmed using the recordings obtained from the wearable cameras (Figure 3a–j). Behaviors related to postures, standing and lying, were easily distinguished from each other in the footage by the distance from the ground (Figure 3a,b). Eating hay or concentrate and drinking were easily differentiated by visually observing what calves were ingesting on the footage (Figure 3c–e). Licking around the mouth, grooming another calf, self-grooming, and licking and biting objects could be distinguished because the calves’ tongues were well recorded, and the observer could see what the calves were doing with their tongues (Figure 3f–j).

### 3.2. Comparison of Direct Observations and Observations of Images Obtained from a Wearable Camera

**Figure 4:**
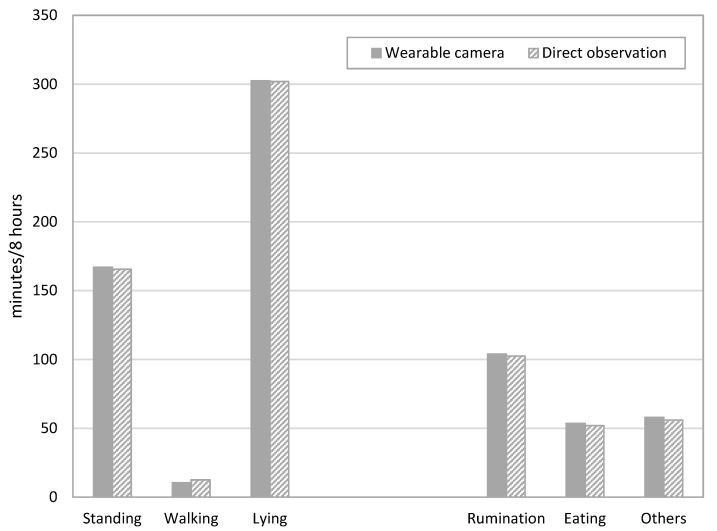
Total observation time for the different calf postures and behaviors, and the corresponding time between direct and wearable camera observations during the study investigating the suitability of wearable cameras for monitoring juvenile bovine behavior.

Figure 4 shows the actions and the corresponding times for each direct and wearable camera observations. Direct observations were made from 11:00 to 12:00 and 14:00 to 15:00 for each calf, making the total observation time for all calves 8 h.

For the posture, lying was observed for 302 min (62.9% of the total 8 h of observation time) by direct observation and 302.5 min (63.0% of the total 8 h of observation time) using the wearable camera.

Rumination was observed for 102.5 min (21.4% of the total 8 h of observation time) by direct observation and 104 min (21.7% of the total 8 h of observation time) using the wearable camera.

Eating occurred for 52 min (10.8% of the total observation time) by direct observation and for 53.5 min (11.1% of the total observation time) using the wearable camera, which was almost the same time as for other behaviors.

Table 4 and Table 5 show the differences between wearable camera observation and direct observation per behavior for each calf in minutes. Table 4 is for posture (standing, moving, and lying) and Table 5 is for behavior (rumination, eating, and others).

The interrater reliability (Cohen’s kappa coefficient) between direct and video observations is shown in Table 6. The coefficients between the direct observation and videos from wearable cameras for the postures and behaviors were significant in each animal (*p* < 0.01).

Table 7 shows the behaviors with nonmatching results and the times for which each observation method was compared.

The difference in time for standing and moving observations was 7.5 min, which is the largest difference in posture. However, the differences between standing vs. lying and moving vs. lying were small.

## 4. Discussion

As seen in the example photographs taken from the video (Figure 3), the right side of the calf’s mouth (hereafter referred to as the mouth) was recorded on the left side of the screen, and the background was recorded on the right side. By comparing the backgrounds in Figure 3a,b, it was easy to distinguish standing from lying down because there was a greater distance to the ground when standing and a shorter distance when lying down. Ruminating behavior could be distinguished because the calf’s mouth moved, and the background shook constantly. In the feeding behavior, the feed ingested was recorded in the background; therefore, it was possible to identify this behavior by observing the feeding target (Figure 3c,d). The behavior of drinking water was confirmed from the movement of the mouth and the water tank recorded in the background (Figure 3e). The behavior of licking water with the tongue was also observed. Under direct observation, it was difficult to ascertain whether drinking behavior occurred because this behavior could only be observed by the animal placing its mouth in the water trough. Behaviors such as licking and biting could be easily registered based on the recorded observations (Figure 3f–j) because licking and biting targets were recorded clearly.

Rumination was confirmed to be a behavior suitable for observation with a wearable camera. Moreover, a recording obtained by a wearable camera can be paused and replayed from any point and reviewed indefinitely; thus, rumination was less likely to be overlooked using wearable camera observations compared to direct observation. Furthermore, rumination was easy to distinguish from the camera recordings as there were few movements other than the mouth, and it represented a continuous behavior with a long duration. Moreover, the calves were often observed lying, which is consistent with previous research, including that of Morita et al. [33].

Furthermore, there was some difference between standing and moving observations. The difference occurred because the movement was defined as limb movement (Table 2) and limb movement cannot be seen in the image obtained with the wearable camera. Consequently, the behavior was missed and the animal was considered to be standing. In addition, standing was misclassified as moving in two cases because the animal’s head shook considerably, although it was not accompanied by limb movement. If the duration of the movement was long, the movement could be identified by a change in the background of the image. The background of the barn where this experiment was conducted was monotonous, and the floor area was limited to 58.5 m^2^, which further complicated the distinction between standing and moving. If the movement range was wide, such as that in a pasture, and the background of the image changed considerably, or if the movement had been defined as walking with one complete stride or more, the accuracy for the movement observation based on a wearable camera recording would have been better.

Although behaviors can be grasped by a wearable camera, the following points should be noted from the viewpoint of welfare of calves. Welfare should always be monitored by a human at regular intervals and should never be replaced by wearable cameras. For the wearable cameras used in our experiment, there is an entrapment risk as the halter expands around the cameras. Therefore, considering their welfare, calves need to be regularly welfare-checked by an individual.

Further studies are required for testing a larger sample size, with multiple observation periods and human observers. In addition, if wearable cameras having higher performance and smaller sizes are to be sold, testing of such state-of-the-art technology will be necessary.

## 5. Conclusions

Comparing behavioral observations obtained by direct observation with those obtained through a recorded video using a wearable camera indicates that it is possible to observe calf behavior using a wearable camera. Postures such as standing and lying and behaviors such as feeding and rumination could be observed as accurately as through direct observations. During direct observations, behaviors were sometimes difficult to observe owing to the position of the observer and the structure of the barn; however, with a wearable camera, the mouth of the calf was always recorded, and the mounting position (halter) of the wearable camera was suitable for observing behaviors around the mouth. These results show that recorded footage can be used for automatic behavioral observation using AI because the video can record the targeted behavior to the exact degree of accuracy as obtained by direct visual observation. This was a preliminary study; however, it shows the potential uses of this technology and highlights many areas for its application.

## Figures and Tables

**Table 1:** Birth date, body weight, weaning date, and observation date of the female Holstein calves monitored during the study to investigate the suitability of wearable cameras for observing juvenile bovine behavior.

Calf No.	Birth Date	Body Weight ^※^ (kg)	Weaning Date	Observation Date
876	15 October 2017	86.4	23 December 2017	25 December 2017
877	18 October 2017	86.2	23 December 2017	22 December 2017
878	21 October 2017	84.8	25 December 2017	26 December 2017
879	27 October 2017	81.0	26 December 2017	27 December 2017

^※^ At the start of the experiment (18 December 2017).

**Table 2:** Definitions for the postures observed during the study investigating the suitability of wearable cameras for monitoring juvenile bovine behavior.

Posture	Definition
Standing	Standing without moving legs.
Moving	Moving the leg, including taking one step (twitches in the leg or sliding on the floor).
Lying	Lying with the sternum in contact with the ground or flat on the side.

**Table 3:** Definitions for the behaviors observed during the study investigating the suitability of wearable cameras for monitoring juvenile bovine behavior.

Behavior	Definition
Rumination	Displaying behaviors associated with rumination (e.g., regurgitating, chewing, and swallowing)
Feeding	Taking feed (hay or concentrate) in mouth, including chewing hay or concentrate beside hay rack or feed trough. For unweaned calf, milk feeding using an automatic feeder is included.
Others	Drinking, self-grooming, grooming another calf, licking, or biting objects.

**Table 4:** Differences between wearable camera observation and direct observation per posture for each calf.

Calf No. 876		Direct Observation		
		Standing	Moving	Lying
Wearable camera	Standing	94	1	3
	Moving	4	5	0
	Lying	0	1	132
**Calf No. 877**		**Direct Observation**		
		**Standing**	**Moving**	**Lying**
Wearable camera	Standing	48	0	0
	Moving	1	3	0
	Lying	0	0	188
**Calf No. 878**		**Direct Observation**		
		**Standing**	**Moving**	**Lying**
Wearable camera	Standing	62	0	1
	Moving	0	6	0
	Lying	0	0	171
**Calf No. 879**		**Direct Observation**		
		**Standing**	**Moving**	**Lying**
Wearable camera	Standing	120	0	5
	Moving	1	1	0
	Lying	1	0	112

**Table 5:** Differences between wearable camera observation and direct observation per behavior for each calf.

Calf No. 876		Direct Observation		
		Rumination	Eating	Others
Wearable camera	Rumination	55	0	0
	Eating	0	29	0
	Others	0	0	20
**Calf No. 877**		**Direct Observation**		
		**Rumination**	**Eating**	**Others**
Wearable camera	Rumination	20	0	0
	Eating	0	33	0
	Others	0	0	18
**Calf No. 878**		**Direct Observation**		
		**Rumination**	**Eating**	**Others**
Wearable camera	Rumination	99	0	0
	Eating	0	20	0
	Others	0	0	19
**Calf No. 879**		**Direct Observation**		
		**Rumination**	**Eating**	**Others**
Wearable camera	Rumination	30	0	0
	Eating	0	13	0
	Others	0	0	31

**Table 6:** Cohen’s kappa coefficient for each calf by posture (standing, moving, and lying) and behavior (rumination, eating, and others).

Calf No.	Posture	Behavior
876	0.93 ***	1.00 ***
877	0.99 ***	1.00 ***
878	0.99 ***	1.00 ***
879	0.94 ***	1.00 ***

***: *p* < 0.01.

**Table 7:** Differences in the time recorded for different postures based on direct and wearable camera observations during the study investigating the suitability of wearable cameras for monitoring juvenile bovine behavior.

Direct Observation	Wearable Camera	Time (min)	Sum (min)
Standing	Moving	3.0	7.5
Moving	Standing	4.5	
Standing	Lying	0.5	1.0
Lying	Standing	0.5	
Moving	Lying	0.5	0.5
